# Outbreak Breakthrough: Using Whole-Genome Sequencing to Control Hospital Infection

**DOI:** 10.1289/ehp.123-A281

**Published:** 2015-11-01

**Authors:** Carrie Arnold

**Affiliations:** Carrie Arnold is a freelance science writer living in Virginia. Her work has appeared in *Scientific American*, *Discover*, *New Scientist*, *Smithsonian*, and more.

The British soldier on the trauma and burns ward at Queen Elizabeth Hospital Birmingham brought home more than his injuries when he was evacuated from Afghanistan in July 2011. Like many wounded veterans,^1^ he also carried an opportunistic pathogen called *Acinetobacter baumannii* that was resistant to numerous classes of antimicrobials. If this specific strain of bacteria spread to others in the hospital, the doctors there would have few, if any, options for treating their patients. Keeping the bacterium contained through vigorous infection control procedures seemed the only hope.

**Figure:**
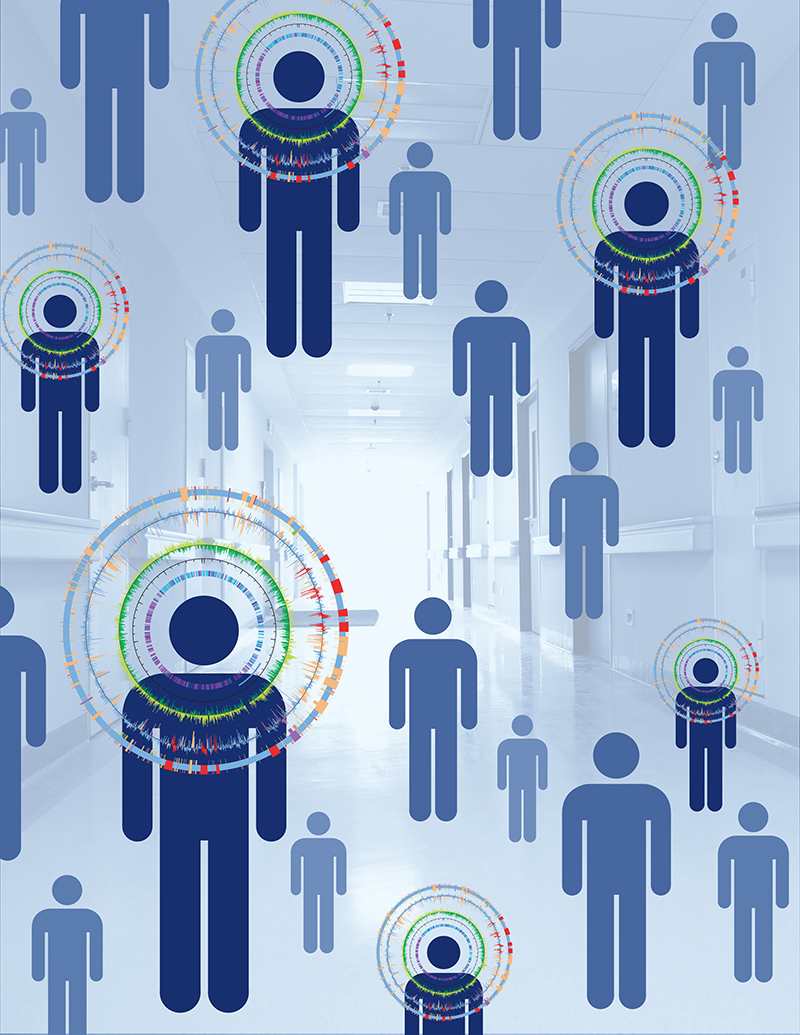
The level of detail provided by whole-genome sequencing could give hospitals the tools they need to stop outbreaks before they start. Background: © hxdbzxy/Shutterstock; E. coli O157:H7 genome map reprinted by permission from Macmillan Publishers Ltd: doi:10.5923/s.microbiology.201401.02

After a week, another patient developed symptoms of infection with *A. baumannii*. Basic DNA analysis in the hospital’s clinical laboratory showed the strain had the same specific molecular pattern as the soldier’s. Soon, other patients throughout the hospital began falling ill with the same infection. Physicians considered the possibility of “cryptic transmissions,” in which a person can acquire the bacterium from a carrier who isn’t even sick. But even enhanced infection control procedures such as deep cleaning and restrictions on visitors weren’t stopping the outbreak, leading hospital officials to conclude the pathogen likely was being transmitted through the environment rather than directly from person to person. If they could identify the environmental source of the infection, they could stop the outbreak.

The problem was that older DNA analysis techniques did not provide enough detail on the bacteria involved to track transmission. All that officials could say with any degree of certainty was that all the affected individuals were likely part of the same outbreak. So the hospital sought advice from microbial geneticist Mark Pallen, now at the University of Warwick, who used an approach known as whole-genome sequencing (WGS) to sequence the genomes of 114 *A. baumannii* samples isolated from patients. By mapping the epidemiology of the WGS data, Pallen and colleagues were able to identify the environmental sources of transmission.^2^

What was most striking to Pallen was that most of the patients who tested positive for *A. baumannii* had been treated in the hospital’s operating theaters. One theater in particular was implicated in the infections of three different patients, which was confirmed by WGS of environmental and patient isolates. The operating theater was closed for deep cleaning, and the outbreak seemed over.

But after a lull of six weeks, a new case turned up, which WGS linked to the original *A. baumannii*. With no other infected patients in the hospital, Pallen took a closer look at potential environmental sources. The researchers eventually identified a special bed for burn patients that had been used by a previous infected patient. A second round of deep cleaning finally stopped transmission for good.

Today, as the cost and time required to sequence genomes has plummeted, more hospitals and health-care agencies are turning to WGS to study disease outbreaks, and the method promises to revolutionize standard methods of infection control and contact tracing during outbreaks.^3^ Eventually, experts say, WGS likely will be used not only to track outbreaks of pathogens but also to catalog the microbes present in a given environment and the ways in which patients and staff move these microbes from place to place, ward to ward, and person to person. With this knowledge, hospitals may even be able to stop outbreaks before they start.

## No Time to Waste

Basic hospital infection control measures, such as disinfecting surfaces and ensuring doctors and nurses wash their hands between patients, dramatically lower infection rates. But bacteria can survive for long periods, even after vigorous disinfection procedures,^4^ and WGS analysis has shown they are able to travel via pathways no one had predicted.^5^ Although these transportation events and hiding spots might go unseen, researchers hope that looking directly at the bacterial genomes may reveal bacterial strain circulation.^6^

The first organism to have its entire genome sequenced was the common respiratory pathogen *Haemophilus influenzae*, a procedure first reported in 1995^7^ that cost around $1 million and took more than a year.^8^ At the same time, scientists began moving toward ways of identifying bacteria using their DNA, even if it didn’t involve identifying every last nucleotide. In one method, pulsed-field gel electrophoresis (PFGE), the bacterial genome is chopped into small pieces using enzymes that split DNA near certain nucleotide sequences known as restriction sites. The fragments are then separated by size, and the pattern of fragments creates a unique fingerprint for different types of bacteria. Other methods sequence only certain parts of the bacterial genome, which lets researchers identify different aspects of the pathogen they are looking at, such as species and genetic markers that could help determine which bacteria were part of which outbreak—something that isn’t always possible with PFGE.

“Older methods of investigating outbreaks were done by methods that were, by comparison [with WGS], incredibly crude,” says William Hanage, a microbiologist at Harvard University. “You would chop up the DNA … and you’d get a banding pattern on a gel. And you’d hold that up to the light and squint at it and say whether two isolates had the same banding pattern.”

In contrast, genomes provide much more detail and enable much more precision in determining which pathogens are being transmitted and how it is happening. “Genomics is going to revolutionize infection control,” Hanage says. “We’re going to have genome sequencing of isolates as a matter of course.”

The first automated gene sequencers used a method developed by British biochemist Frederick Sanger, and, by the late 1990s, could sequence 2.88 million bases per day in sections up to 900 base pairs long.^9^ WGS—also referred to as high-throughput or next-generation sequencing—first became commercially available in 2005 and could sequence many genomes at the same time.^10^ These systems could sequence only shorter sections of DNA that had to be laboriously pieced back together into a full genome, but they had the advantage of allowing more sequencing at a lower cost.^11^

Despite these improvements, the process was still relatively slow. “In the early stages of next-generation sequencing technology it would take ten to fourteen days from a patient having an infection to getting a genome sequenced, and that’s not fast enough for infection control,” says University of Michigan microbiologist Evan Snitkin. Today, Snitkin says, the newest technology can turn around results in a matter of hours. And while many investigators still culture the samples they collect before performing WGS, there is evidence that clinical samples can be tested directly and reliably.^12^

**Figure:**
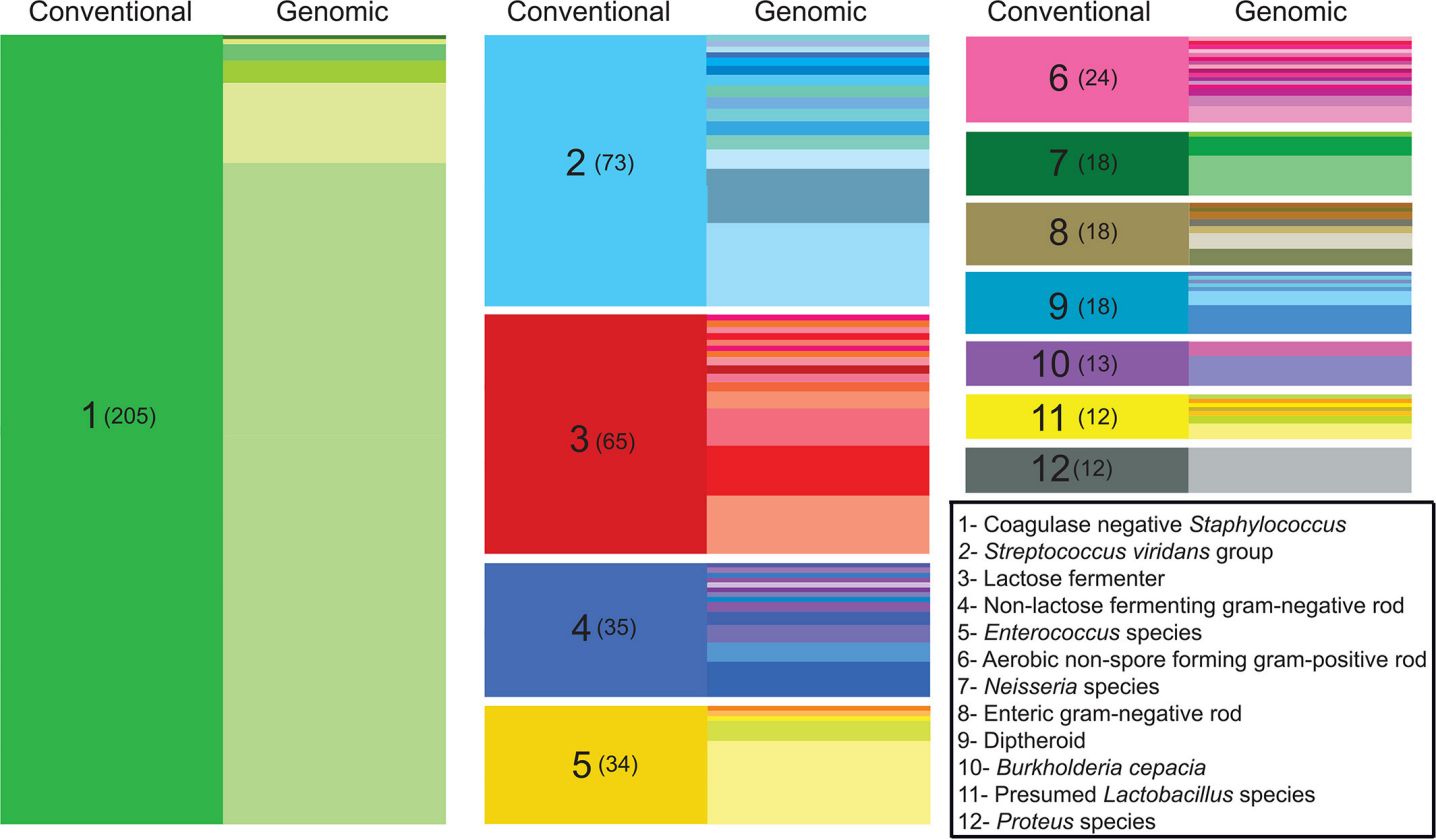
Researchers conducted prospective surveillance of the bacteria found in one hospital’s intensive care units over the course of a year.^28^ WGS was performed on 1,229 bacterial isolates, revealing a number of novel bacterial species. Compared with conventional classification of species by phenotypic or biochemical qualities (indicated on the left side of each column above), WGS enables more detailed species-level classification (right side of each column). Source: Roach et al. (2015)^28^

## Moving into Real Time

Before the outbreak at Queen Elizabeth Hospital Birmingham, WGS was often used as a way to understand an outbreak after it had already happened. In 2010 officials at the British Columbia Centre for Disease Control asked bioinformatician Jennifer Gardy to sequence the genomes of 32 bacterial isolates from a large tuberculosis outbreak in the province. She combined the genome sequences with social-network data that tracked who had come into contact with infected people. With the help of a team including nurses and doctors on the frontline of the outbreak, she was able to reconstruct how the outbreak spread.^13^ “Our group was the first to actually use this technique to reconstruct a near-complete outbreak,” Gardy says.

It wasn’t long before investigators began using WGS to study outbreaks occurring within hospitals themselves. Elimination of hospital-acquired (nosocomial) infections is a priority of the U.S. Department of Health and Human Services.^14^ In 2011 an estimated 722,000 infections were acquired in U.S. hospitals,^15^ and each year hundreds of millions of infections occur worldwide.^16^ Several months after Gardy published her findings, Snitkin tackled an ongoing outbreak of carbapenem-resistant *Klebsiella pneumoniae* at the National Institutes of Health Clinical Center. The outbreak began with a patient in the intensive care unit. As in the *A. baumannii* outbreak at Queen Elizabeth Hospital Birmingham, strict infection control measures were taken, and once again, the organism was contained only temporarily before other patients became ill. Of the 18 people ultimately colonized, 11 died.^17^

When the second patient became ill three weeks after the first patient had been discharged, Snitkin’s team began routine sampling of all patients in the intensive care unit, even if they did not have the symptoms typically associated with *Klebsiella*.^18^ They identified several asymptomatic individuals who were colonized by the bacterium. Then investigation also found the outbreak strain in several sink drains and on a ventilator.

WGS allowed the investigators and infection control officials to reconstruct the outbreak. Because the genome of *K. pneumoniae* mutates so rapidly, Snitkin could track transmission by mapping the evolution of the pathogen over time. “Sequencing isolates from a set of patients can tell you that there is an outbreak, because the isolates are so closely related,” Snitkin says. “In turn, finding a similarly closely related isolate on a common piece of medical equipment could suggest that this piece of equipment is a vector carrying the bacteria between patients.” Having those links gave epidemiologists the evidence they needed to target cleaning and other infection control procedures to stop transmission.

The first report of researchers using WGS to stop an outbreak while it was still happening was published in 2012.^19^ A group in England, headed by University of Cambridge microbiologist Sharon Peacock, was studying an outbreak of methicillin-resistant *Staphylococcus aureus* (MRSA) in a neonatal intensive care unit at Cambridge’s Rosie Hospital. Their investigation began after three babies tested positive for MRSA within days of each other. Preliminary tests revealed an identical pattern of antimicrobial resistance, indicating the infections were likely linked. When another baby became ill with the same strain of MRSA just days after the unit was deep-cleaned and sterilized, Peacock began collecting environmental swab samples plus blood samples from workers in the Rosie neonatal intensive care unit and families of the infants. Her team sequenced the bacteria in these samples, and she also began a retrospective analysis of MRSA samples isolated over the past six months in the unit.

The sequencing data showed that one worker in the unit carried the same strain of MRSA that was implicated in the outbreak, making her the likely source of ongoing transmission; however, since she had no symptoms, she had no idea she was infected. Treating her with appropriate antibiotics stopped transmission. In all, the group identified more than a dozen infected individuals, including six babies, who developed severe infections requiring treatment.

**Figure:**
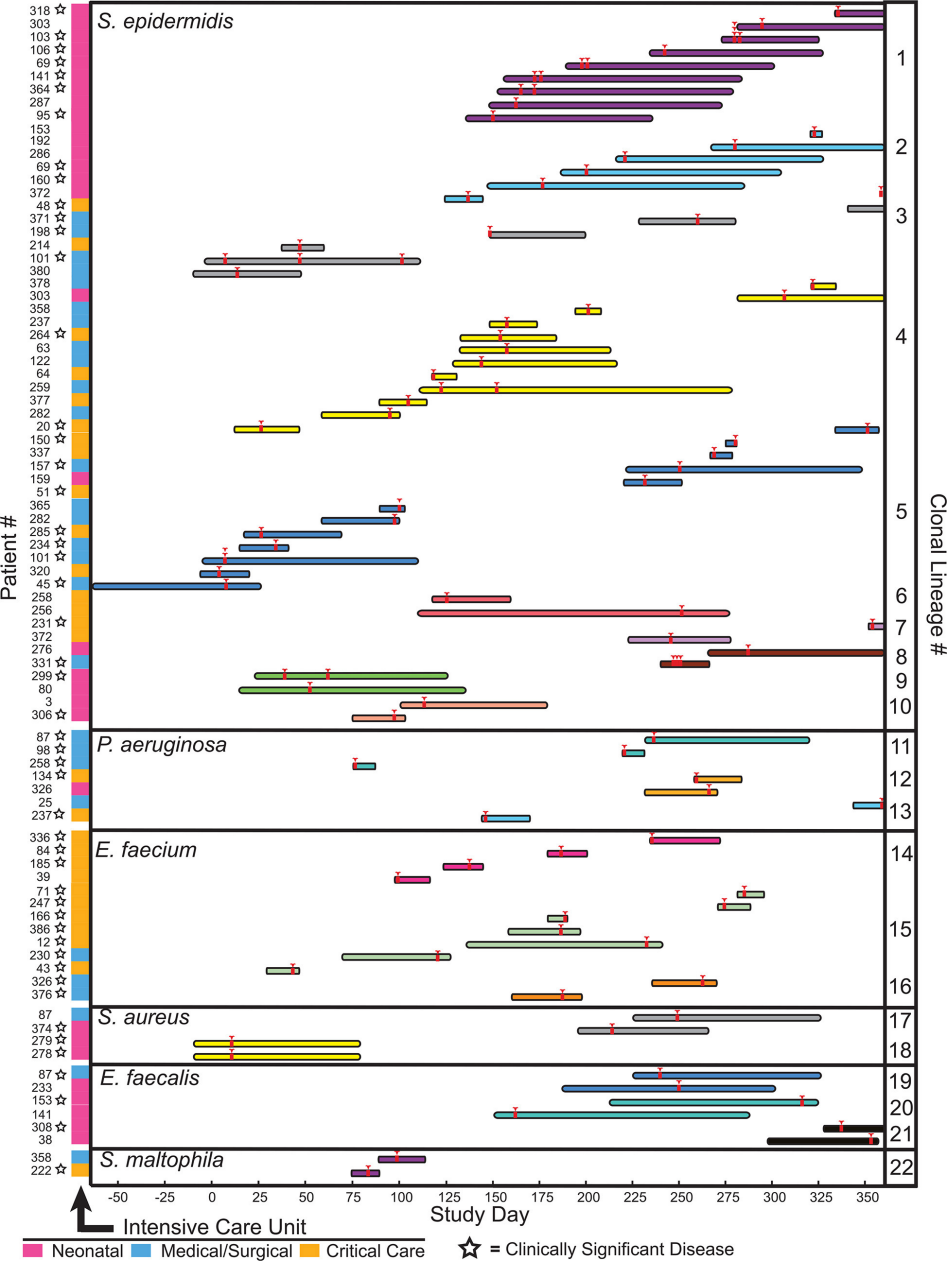
In their year-long surveillance,^28^ Stephen Salipante and colleagues demonstrated how bacterial clones (lineages of bacteria traceable to a single ancestor) moved between different intensive care units over time. They identified 22 distinct clones that were shared by multiple patients, each shown here in a separate color. The length of each bar indicates how long the patient was in the hospital, while the red arrowheads show when sampling occurred. Source: Roach et al. (2015)^28^

## Gaining Ground

More and more laboratories around the world have begun to tackle hospital outbreaks with WGS. In England, University of Birmingham microbial geneticist Nicholas Loman and his staff recently applied WGS to a *Salmonella* outbreak at Heartlands Hospital, ultimately tracing the source of the outbreak to a broader outbreak affecting other parts of the United Kingdom and Europe.^20^ “Ideally, we will be able to analyze the isolates in the context of everyone else’s data to see what’s going on at a larger scale, and we want to do it on a very rapid time scale,” Loman says.

At other hospitals researchers have studied antimicrobial-resistant outbreaks of *K. pneumoniae*,^21^^,^^22^ *P. aeruginosa*,^23^ *Vibrio cholerae*,^24^ and *Enterobacter cloacae.*^25^ The decreasing size and expense of DNA sequencers has also enabled the use of WGS in less affluent health-care settings. Loman’s graduate student Joshua Quick recently returned from Guinea, where he spearheaded a program to sequence the genomes of all Ebola virus samples isolated at local hospitals as a way of mapping virus transmission. And University of Oxford microbiologist Stephen Baker used WGS to investigate an outbreak of antimicrobial-resistant *K. pneumoniae* that struck the pediatric wards at a Kathmandu hospital, killing 75% of the affected children.

Sequencing of previously collected samples showed that the bacteria had been transmitted around the Nepali hospital for at least six months before hospital officials became aware of the outbreak. Baker showed that after the bacterium first arrived, various evolutionary factors led to the acquisition of more virulence genes and multidrug resistance, which is what led to such a deadly outbreak.^26^ “We would never have known that this specific *Klebsiella* strain was such a recurring problem without that sequencing data,” Baker says.

Baker’s results, which showed that the bacteria responsible for an outbreak can arrive in a hospital long before the outbreak occurs, support the idea that WGS can play an important role in routine surveillance, says University of Birmingham’s Loman. “These infections don’t come from nowhere,” he says. “Genomic surveillance can identify potential outbreaks before hospitals are even aware of the problem.”

Loman points to a recent study of his in which he sequenced *P. aeruginosa* isolates from environmental samples and burn patients at Queen Elizabeth Hospital Birmingham. Of the 141 isolates sequenced, Loman and his graduate student Quick identified several patients who had *Pseudomonas* infections genetically identical to bacteria found on water taps and showerheads. This route of transmission was known to exist, but it hadn’t been documented in that hospital before.^27^

Nearly halfway around the world, microbiologist Stephen Salipante and colleagues at the University of Washington in Seattle used a similar technique to prospectively survey all the bacteria recovered from the intensive care units of a hospital over the course of a year. Not only did Salipante’s team discover infections caused by novel bacterial species, they also identified a surprising number of cryptic transmissions by asymptomatic individuals. Two-thirds of the recovered isolates were associated with clinically significant infection in patients.^28^

However, adds Salipante, it is unclear whether transmission had occurred in the hospital or whether patients had been exposed prior to admission to bacteria that were endemic in the community. It is also unclear whether the bacteria caused the disease or were just associated with it. “We were really surprised at the intrapatient sharing of isolates, and we don’t really know where these transmissions originate,” he says.

## Toward Clinical Use

Substantial difficulties remain with performing WGS on bacterial samples on a routine basis or even just during outbreaks. The problem, experts say, is not with the sequencing itself but rather with data storage and interpretation. To date, essentially all the examples of using WGS to analyze hospital outbreaks have been conducted in collaboration with academic researchers who help hospital infection control experts interpret the voluminous data that are rapidly produced by sequencers.^29^

Researchers also still need to identify ways to integrate WGS into a clinical laboratory workflow. “Lots of people are thinking about bacterial sequencing, but they don’t know how to move it into routine use,” Loman says.

Regardless of precisely how hospitals decide to track the pathogens spreading around their wards, it’s become clear that, in the very near future, they will be able to do so more quickly than ever. “It’s been amazing to watch—the idea has taken off in a way I never would have predicted, and it provides a level of detail that has never been achieved,” Gardy says. “This isn’t the wave of the future. It’s happening now.”
